# Total absorption in asymmetric hyperbolic media

**DOI:** 10.1038/srep02662

**Published:** 2013-09-16

**Authors:** Igor S. Nefedov, Constantinos A. Valagiannopoulos, Seed M. Hashemi, Evgeny I. Nefedov

**Affiliations:** 1 Aalto University, Department of Radio Science and Engineering, SMARAD Center of Excellence, P.O. Box 13000, FI-00076 Aalto, Finland; 2 Iran University of Science and Technology, Department of Electrical Engineering Tehran 1684613114, Iran; 3 Department of Automation and System Technology, School of Electrical Engineering, Aalto University

## Abstract

Finite-thickness slabs of hyperbolic media with tilted optical axes exhibit asymmetry properties for waves propagating upward and downward with respect to slab interfaces. Under certain conditions, asymmetric hyperbolic media acquire extreme permittivity parameters and the difference between upward and downward propagating waves becomes very large. Furthermore, both waves can be perfectly matched with the free space; such a feature makes possible the development of optically ultra thin perfect absorbers. The proposed approach is unified and allows the use of different 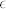-negative materials. Of particular interest is the asymmetric hyperbolic medium, made of silicon nanowires, since it can be directly applicable to solar cell systems.

Absorbers of electromagnetic radiation find wide applications in spectral ranges from radio frequencies to X-rays. One of the first microwave absorbers, consisted of a 120*π*Ω resistive sheet placed over a metal plate at a quarter wavelength distance, was patented by Salisbury in 1952^1^. Numerous solutions for design of absorbers were proposed since that time. Recent advances in development of absorbers became possible by employing low-dimensional carbon nanomaterials and metamaterials - artificial substances with engineered properties. In particular, it is known that a sheet of graphene absorbs about 2.3% of light radiation with a very small reflection in the optical range^2^. Thus, a multi-layered graphene slab could function well as absorber and its performance can be further enhanced with a patterned sheet of doped graphene^3^. Almost perfect absorption in vertically aligned single-walled carbon nanotubes has been also reported in^4^. Saturable absorbers, based on graphene and carbon nanotubes, can be additionally used in mode-locked fiber lasers^5^,^6^,^7^. Finally, total absorption in optically thin layers consisting of arrays of tilted metallic carbon nanotubes was described in^8^.

Application of metamaterials (MM) makes possible the design of resonant elements combining electric and magnetic properties^9^,^10^. There are three beneficial characteristics when one utilizes MMs. Firstly, the geometrical scalability of MMs allows the use similar resonant structures for different parts of the electromagnetic spectrum. Secondly, the possibility to manipulate the magnetic and the electric resonances independently makes feasible to absorb both electric and magnetic fields. Finally, considering MMs in terms of the effective 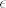 and *μ*, they can provide the impedance matching of absorbing structures with free space. Perfect absorption in metamaterials has been demonstrated in far-infrared^11^, mid-infrared^12^, near-infrared^13^ and visible^14^ ranges. Last decade *hyperbolic metamaterials* have attracted significant scientific interests due to their extraordinary properties. The most remarkable of them is the hyperbolic shape of isofrequency surfaces in the space of wave vectors for eigenwaves propagating in such materials. These surfaces are open and extend to infinity. Hence waves with any large wave vectors can propagate. The hyperbolic-type dispersion is inherent in uniaxial materials in which the axial and tangential components of the permittivity and/or permeability tensors have different signs, referred to as *indefinite metamaterials* by Smith and Schurig in 2003^15^. Some general wave properties of hyperbolic media, such as power, radiated by a point source, were discussed in Ref. 16. Hyperbolic metamaterials offer new possibilities for the sub-wavelength imaging^17^,^18^, the control of spontaneous emission^19^,^20^, the thermal radiative heat transfer^21^,^22^,^23^,^24^. Possible application of corrugated surfaces of *hyperbolic metamaterials* for the radiation absorption was reported by E. Narimanov et al. in^25^.

In the present paper, we propose the concept of *asymmetric hyperbolic media* (AHM) and demonstrate new possibilities offered by AHM for light absorbing. Asymmetry appears as a difference in normal components of waves, propagating downward and upward with respect to AHM interfaces. Asymmetric properties exhibit any uniaxial crystal slabs with optical axes tilted with respect to interfaces. However, new effects, potentially prospective for creation of a new type of optical absorbers, appear in the asymmetric hyperbolic media.

## Results

### Configuration description

**Figure 1:**
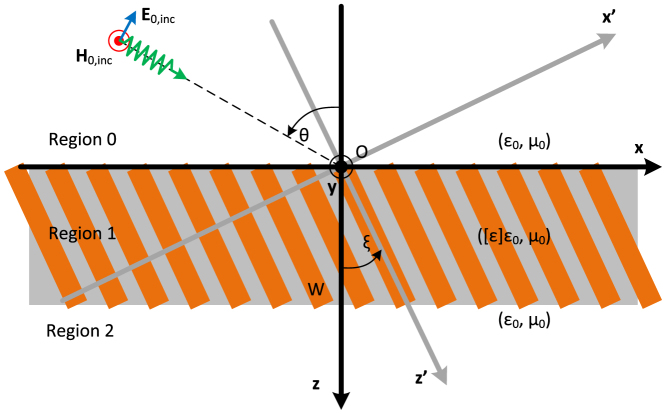
Schematic view of an indefinite medium slab with a tilted optical axis. The *z*′ axis is parallel to the anisotropy axis of MM, the *z* axis in orthogonal to slab interfaces.

A schematic view of a AHM slab is shown in Fig. 1, where two alternative Cartesian coordinate systems, primed: (*x*′, *y*′, *z*′) and unprimed: (*x*, *y*, *z*), are also defined. The optical axis is tilted by the angle *ξ* with respect to slab interfaces. The structure is illuminated from above with an electromagnetic wave that possesses a single magnetic component parallel to **y** axis (TM polarized) propagating along direction forming an angle *θ* with **z** axis. For eigenwaves propagating in AHM we take the space-time dependence of fields as exp [−*i*(*ωt* − *k_x_x* − *k_z_z*)]. The transverse component of wave vector equals to *k_x_* = *k*_0_ sin *θ*, where *k*_0_ is the wavenumber in free space. The relative permittivity tensor [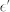] is diagonal when expressed in the coordinate system (*x*′, *y*′, *z*′). The slab has a thickness *W* and is made of plasmonic anisotropic material so that 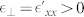, 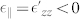; accordingly, the polar axis of a hyperbolic isofrequency is directed along the *z*′ axis. The dispersion relation for eigenwaves in this uniaxial crystal reads 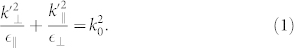
In the unprimed (*x*, *y*, *z*) coordinate system, the relative permittivity tensor 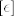 becomes non-diagonal and the normal component of the wave vector is given by 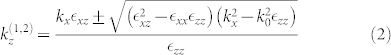
(expressions for the tensor 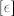 components 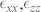, and 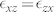 are given in Online Supplementary Information). Here the solution 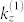 corresponds to the sign “+” in formula (2) and 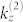 to the sign “−”. In order to recognize what kind of solution corresponds to the wave, excited at the interface by the incident wave, we should examine imaginary parts of 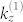 and 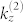. According to the chosen time-space dependence, the imaginary part of *k_z_* has to be positive for the downward wave. It is remarkable, that the normal wave vector components are different for waves, propagating upward (towards smaller *z*, 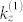) and downward (towards larger *z*, 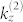) with respect to interfaces.

### Interesting case

The following combination of parameters is of great interest 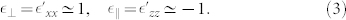
One can show (see Online Supplementary Information) that such a medium acquires extreme properties if 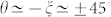. More specifically, the permittivities in the coordinate system (*x*, *y*, *z*) are evaluated as: 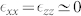, 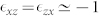. In such a case, the difference between 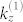 and 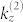 becomes extremal; in particular, 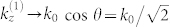, as in free space, and 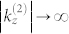.

Another remarkable feature of this medium concerns the transverse wave impedances for the two waves (upward and downward) excited into the slab and corresponding to the wavenumbers 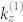 and 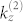. What is obtained is that these two waves despite the substantial difference in their spatial oscillations, are characterized by the same impedance given below: 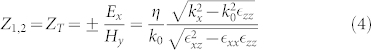
where *η* = 120*π* Ohm. Note that this equality is valid regardless of the choice of the parameters. When additionally *θ* = −*ξ* = ±45° and 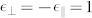, the common impedance of the two waves is the same as that of a plane wave obliquely (under angle *θ*) propagating into free space. Namely: 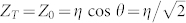. Therefore, for this combination of input parameters, which corresponds to 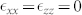 and 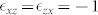, there is a perfect matching at the plane *z* = 0, when the incident field meets the slab structure. In this sense, no reflections are occurred when the incident plane wave enters the asymmetric hyperbolic medium, where the wave with large 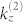 is excited. If the material is lossy, the rapidly oscillating wave would lose the vast portion of its carrying power since, no matter how thin is the slab, the quantity 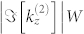 would be substantial. Consequently, the wave would reach almost vanished the bottom surface *z* = *W* and thus the leaked power from the structure would be negligible. In this way, we expect from the device to exhibit substantial absorbing performance.

### Supported wavenumbers

**Figure 2:**
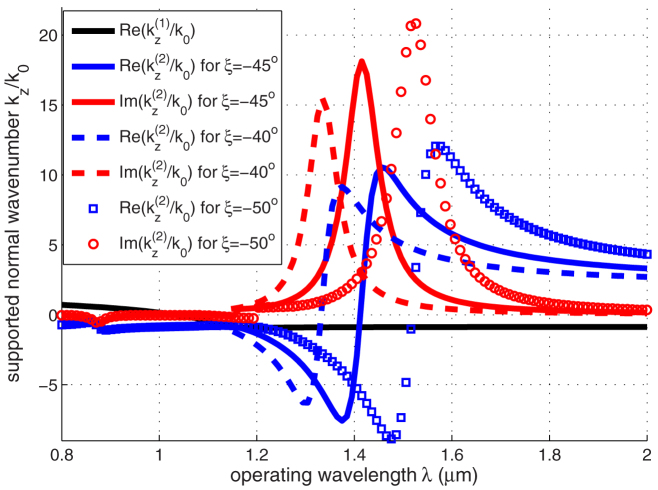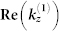 (black), 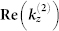 (blue) and 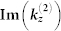 (red). Solid curves correspond to the tilt angle *ξ* = −45°, dashed curves to *ξ* = −40°, and isolated points to *ξ* = −50°.

As an example, we consider eigenwaves, propagating under fixed 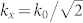 (the incidence angle *θ* = 45°) in a composite of tilted doped silicon nanowires, see Fig. 2. Such Si nanowire composites can be prepared using technologies described in^26^. The permittivity of a doped silicon was calculated using the Masetti model^27^. With concentration of carriers in doped silicon, *N* = 5 × 10^21^ cm^−3^ and volume density of Si nanowires in the composite *p* = 0.1, the conditions (3) are satisfied at the wavelength 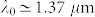, where 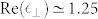, 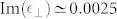, 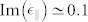. One can see that either real or imaginary part of 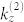 becomes very large in vicinity of *λ*_0_. So, even low material losses would certainly cause high wave attenuation at a short propagation distance under the perfect matching with free space.

### Absorption and reflection

**Figure 3:**
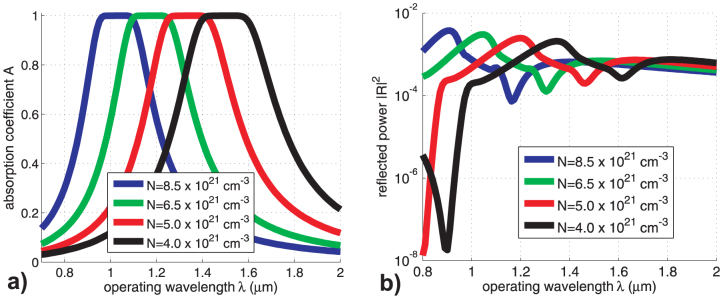
The (a) absorption factor *A* = 1 − |*T*|^2^ − |*R*|^2^ and (b) reflection |*R*|^2^ coefficient magnitudes (in logarithmic scale) as functions of the operating wavelength *λ* for various different concentrations *N*.

Let us consider a plane wave transmission through a slab of tilted Si nanowires. We use the 2 × 2 transfer matrix method, modified for the general case where: 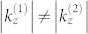, see details in^8^. Wavelength dependence of absorption coefficient *A* (ratio of the absorbed power over the incidence power, taken equal to unity), calculated for different concentration of carriers, is shown in Fig. 3a. The thickness of silicon nanowire layer was taken to be *W* = 200 nm. It is remarkable, that the thickness resonance is absent due to the difference in propagation constants for upward and downward waves within the slab. Furthermore, the best absorption is observed for the tilt angle *ξ* = −40°, despite ideal absorption condition (for *θ* = 45°) correspond to *ξ* = −45°. Such a difference is clearly attributed to the deviation of parameters from the perfect absorption conditions, particularly, because 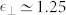. In Fig. 3b, we represent the variation of the squared reflection |*R*|^2^ coefficient of the waves produced by the slab. Due to the unitary magnitude of the obliquely incident plane wave, the normalization condition reads: *A* + |*T*|^2^ + |*R*|^2^ = 1, where *T* is the related transmission coefficient. It is noteworthy that the level of reflections, for the considered case, is extremely small within whole the shown wavelength range from 0.7 *μ*m to 2 *μ*m.

**Figure 4:**
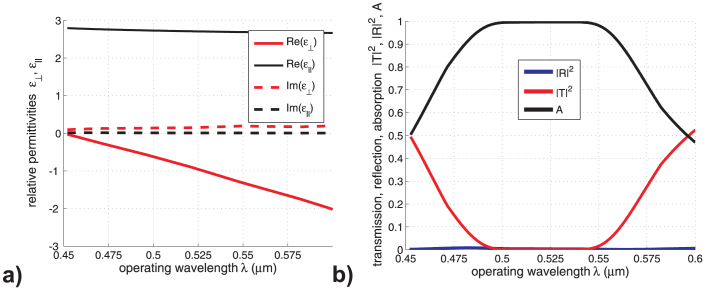
(a) Relative permittivities 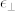, 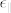 versus operating wavelength. (b) Transmission |*T*|^2^, reflection |*R*|^2^ and absorption *A* coefficients as functions of the operating wavelength *λ* for AHM, made of the silver-dielectric multilayer.

Based on the presented results, one observes that very high concentrations of charge carriers in Si nanowires are necessary for achieving the perfect absorption in the visible range. Such a demand could be raise issues related to the materialization of the structure and the validity of the adopted model. However, the presented graphs are definitely useful for illustration. Moreover, similar effects are recorded for a more realistic plasmonic structure utilizing silver sheets instead of silicon rods, which is deprived of the aforementioned weaknesses. In particular, it is clearly shown in Fig. 4a that hyperbolic properties are also achieved for silver layers, since the transversal and the parallel components of the permittivity tensor are of opposite sign for an extended frequency band. As a result, highly absorbing efficiency is demonstrated in Fig. 4b, where both reflection and transmission are of negligible magnitude.

As far as the real and imaginary parts of the permittivity of silver are concerned, we used interpolated data from^28^. The following parameters of the multilayer are employed: the thickness of Ag layer was taken to be *h_m_* = 2 nm, the thickness of the dielectric layer *h_d_* = 75 nm, the permittivity of dielectric 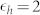, the total thickness of the AHM slab *W* = 60 nm. Formulas for components of the effective permittivity are given in Supplementary Online Materials. We realize that the effective medium approach applied to the periodic structure with the period of lattice *h_m_* + *h_d_* = 77 nm is not applicable to so a small slab thickness, but this example is given for illustration that the total absorption is possible in optically-ultrathin layers of AHM in the visible range. The tilt angle was taken to be *ξ* = −60° and the incidence angle *θ* = 54°.

### Varying incidence and tilt angles

**Figure 5:**
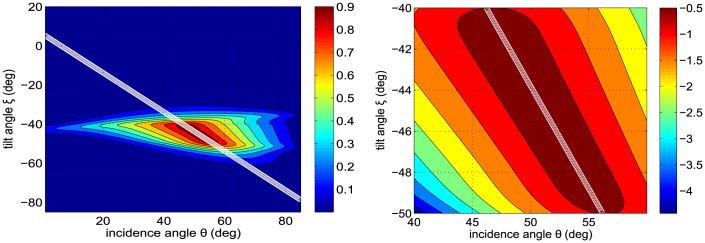
The absorption *A* in contour plot as function of the incidence angle *θ* and tilt angle of the nanorods *ξ* for (a) an extended region in linear scale and (b) the maximum-value region in dB (20 log_10_*A*). The line of the white circles corresponds to the optimal line: *ξ* = −*θ* + 6.2°. Plot parameters: *W* = 200 nm, *λ* = 1.43 *μ*m.

In Fig. 5a we represent the absorption coefficient *A* in a contour plot with respect to the incidence angle *θ* and the tilt angle *ξ*. We can observe a region roughly defined by the inequalities {40° < *θ* < 60°, −50° < *ξ* < −40°} where *A* takes substantial values and one should emphasize on such combinations (*θ*, *ξ*) for further elaboration. It is remarkable that the maxima exist along a straight line which can be approximately defined by the equation: *ξ* = −*θ* + 6.2° which is appeared in Fig. 5a as a sequence of small white circles. Such a formula has been derived after extensive observations for several values of slab thicknesses *W* and operational wavenumbers *λ*. In Fig. 5b, we show the variation of the same quantity in dB 20 log_10_ *A* with respect to the same parameters as above by “zooming” in the interesting region. Our estimation for the straight line of maxima stated above has been further validated and one can clearly see that it constitutes a successful “rule of thumb” for rough computations.

### Numerical simulations

**Figure 6:**
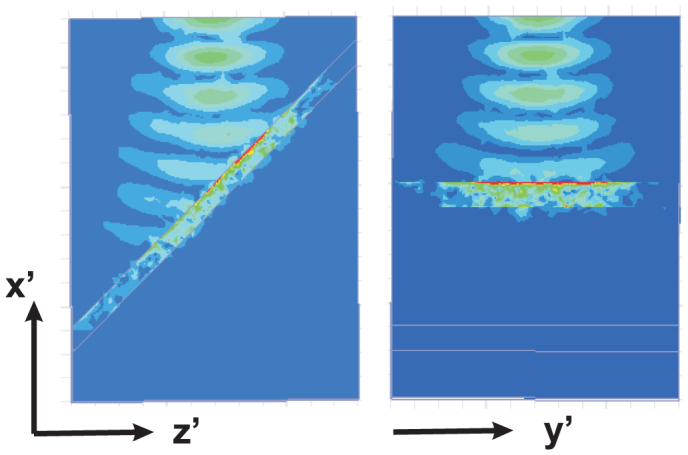
Absorption of the Gaussian beam, shown in the *x*′*z*′ plane (left) and *x*′*y*′ plane (right).

In Fig. 6, we demonstrate the results from ANSOFT HFSS numerical simulations for a Gaussian beam incident onto one tilted absorbing structure. The optical axis is parallel to the *z*′-axis, as in Fig. 1. Narrow Gaussian beam and absorbing boundary conditions were used due to the absence of *x* − *y* symmetry planes for this structure. Simulations were implemented at *λ* = 1.15 *μ*m. A total absorption effect is observed for a slab thickness equal to *W* = 120 nm, namely close to *λ*/10.

**Figure 7:**
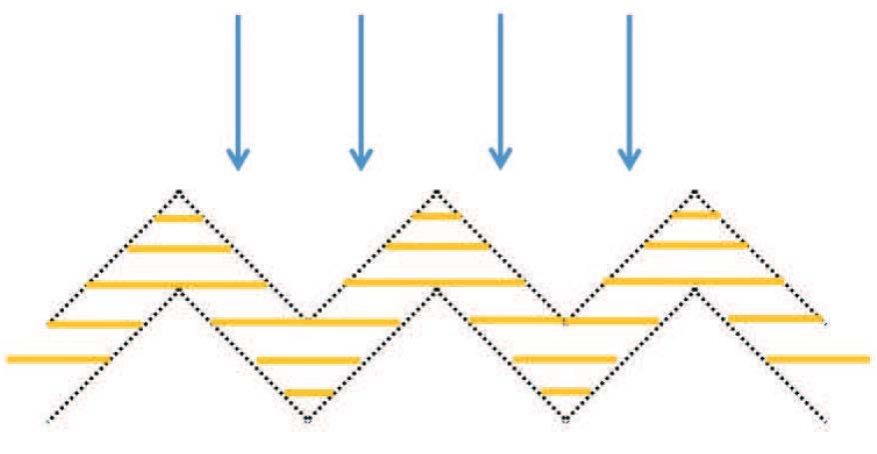
Schematic view of absorbing structure. Blue arrows show illumination direction. Yellow lines show the anisotropy axis direction.

**Figure 8:**
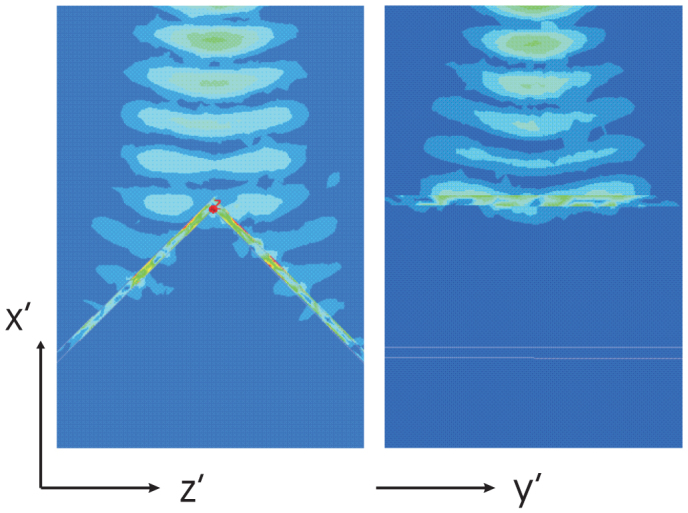
Absorption of the Gaussian beam, shown in the *x*′*z*′ plane (left) and *x*′*y*′ plane (right).

Let us now investigate an alternative absorbing structure with scheme with angles as shown in Fig. 7. The blue arrows show illumination direction, while the yellow lines indicate the anisotropy axis direction. The field density for this specific configuration as obtained form the simulation software is shown in Fig. 8. Wavelength and parameters of the structure are the same as for the planar device. One can see that corners introduce a minor reflection.

## Discussion

In this article we have demonstrated that hyperbolic media with tilted optical axes possess unusual properties and allow realization of materials, supporting propagation of waves, characterized with very large wave vector components while being perfectly matched with free space. Simultaneous fulfillment of these conditions cannot be achieved in any known materials and opens a door for creation of a new class of optically ultra thin absorbers. The proposed approach is universal since different 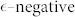 materials can be exploited for its realization; for example silicon and noble metal nanowires, carbon nanotubes, graphene multiple layers, etc can be used instead.

We have found such conditions, that electromagnetic waves, passing through finite-thickness slabs of such media, are characterized by the following intriguing features. Firstly, we observed that the imaginary part of the wave vector component, normal to the interface, becomes very large; accordingly, even a low damping factor of a hyperbolic medium provides full absorption at optically ultra thin distance. Secondly, we showed that there is the perfect impedance matching and a totally eliminated reflection. Thirdly, we noted the interesting feature, which does not relate directly to absorption, that within the slab, with respect to interfaces, can propagate backward waves, see Fig. 2.

## Methods

The employed formulas have been obtained based on the rigorous solution of the Maxwell's equation for the structure depicted in Fig. 1. In particular, the supported waves into the anisotropic slab with relative permittivity tensor 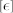 are found by assuming a TM-polarized plane wave with a single magnetic component parallel to the **y** axis, namely: **H** = **y** exp[*i*(*k_x_x* + *k_z_z*)]. To this end, we substitute the latter expression into the vectorial Helmholtz equation: 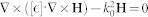 to derive the dispersion equation (1). In this way, the expressions (2) for 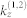 are obtained with *k_x_* = *k*_0_ sin *θ* as dictated by the incident illumination. The two supported waves are identified and therefore one can readily deduce the expression (4) for the respective transverse impedances *Z*_1,2_ (see Online Supplementary Information).

The reflected magnetic field exists into the upper vacuum half space labeled as region 0 possesses a complex amplitude *R* and takes the form: **H**_ref_ = **y** *R* exp[*ik*_0_(sin *θx* − cos *θz*)]. The transmitted magnetic field correspond to the lower vacuum half space (region 2) and is written as: **H**_trans_ = **y***T* exp[*ik*_0_(sin *θx* + cos *θz*)]. After imposing the boundary conditions along the planar surfaces *z* = 0, *W*, the analytical expressions for *R*,*T* are obtained. As stated above, the absorption coefficient is evaluated as: *A* = 1 − |*R*|^2^ − |*T*|^2^. Needless to say that the permittivity tensor 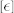 is computed from 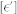, multiplied with the suitable coordinate rotation matrices by angle *ξ* (see Online Supplementary Information).

## Author Contributions

I.S.N. and C.A.V. performed derivation of formulas and wrote manuscript. S.M.H. helped in formulas derivation and calculations. E.I.N. implemented numerical HFSS simulations. All authors reviewed the manuscript.

## Supplementary Material

Supplementary Information Supplementary Info

Supplementary Information Animation for Fig. 6a

Supplementary Information Animation for Fig. 6b

Supplementary Information Animation for Fig. 8a

Supplementary Information Animation for Fig. 8b
