# Supplementary material for: Total absorption in asymmetric hyperbolic media
Source: Sci Rep. 2013 Sep 16;3:2662. doi: 10.1038/srep02662 (PMC3773620; doi:10.1038/srep02662)
Supplement: Supplementary Information — Supplementary Info [file srep02662-s1.pdf]

Supplementary information
Total absorption in asymmetric hyperbolic media

Igor S. Nefedov[1], Constantinos A. Valagiannopoulos[1],
Seed M. Hashemi[2] and Evgeny I. Nefedov[1]
[1]AaltoUniversity,
Department of Radio Science and Engineering, SMARAD Center of Excellence,
P.O. Box 13000, FI-00076 Aalto, Finland
[2]Iran University of Science and Technology,
Department of Electrical Engineering
Tehran 1684613114, Iran
*To whom correspondence should be addressed; E-mail: igor.nefedov@aalto.fi

In this Supplementary Information section, we provide specific details on analytical solution of the plane wave transmission through the hyperbolic medium (HM) slab including the analytical evaluation of the wave impedances at the slab boundaries and the derivation of the elements of the permittivity tensors for the constituent anisotropic material. Furthermore, the models of some concrete HM are presented and the corresponding homogenized permittivities are computed.

1 Permittivity tensor for a hyperbolic medium with a tilted optical axis and eigenvalue equation

In the coordinate system (x', y', z') (see Fig. 1) the relative permittivity tensor of an uniaxial medium reads as

$$[\epsilon'] = \epsilon_{\|}\mathbf{z}'\mathbf{z}' + \epsilon_{\perp}(\mathbf{x}'\mathbf{x}' + \mathbf{y}'\mathbf{y}') \tag{1}$$

where $\epsilon_\perp$ is the transversal component of the permittivity tensor. The permittivity dyadic in the (x, y, z) coordinate system can be expressed through rotation transformation as:

$$[\epsilon] = \overline{\overline{U}} \cdot [\epsilon'] \cdot \overline{\overline{U}}^T \tag{2}$$

where $\overline{\overline{U}}$ is the matrix of rotation around the y axis:

$$\overline{\overline{U}} = \begin{bmatrix} \cos\xi & 0 & -\sin\xi \\ 0 & 1 & 0 \\ \sin\xi & 0 & \cos\xi \end{bmatrix} \tag{3}$$

and $\overline{\overline{U}}^T$ is the transposed matrix. The Cartesian components of $[\epsilon']$ read:

$$\begin{aligned} \epsilon_{xz} &= \epsilon_{zx} = (\epsilon_\perp - \epsilon_\|) \cos\xi \sin\xi \\ \epsilon_{xx} &= \epsilon_\| \sin^2\xi + \epsilon_\perp \cos^2\xi \\ \epsilon_{zz} &= \epsilon_\| \cos^2\xi + \epsilon_\perp \sin^2\xi. \end{aligned} \tag{4}$$

Thus, the permittivity tensor becomes non-diagonal, but symmetric, i.e. $\epsilon_{xz} = \epsilon_{zx}$, in new coordinate system.

For eigenwaves propagating in AHM we take the space-time dependence of fields as $e^{\exp[-i(\omega t - k_x x - k_z z)]}$. Two wave characteristics are needed for solution of any interface boundary-value problem: the normal component of the wave vector k_z and the transverse wave impedance, which is $Z_t = E_x/H_y$ in our case. The transverse component of the wave vector k_x in the HM slab is fixed by the incidence angle θ, $k_x = k_0 \sin\theta$, where k_0 is the wavenumber in free space. To obtain eigenmodes propagating in the structure, we solve source-free Maxwell's equations for TM waves using the constitutive relations for IM with the tilted optical axis and after eliminating the magnetic field, the equation for the electric field (eigenvalue equation) is obtained in the form:

$$\begin{bmatrix} k_z^2 - k_0\epsilon_{xx} & k_x k_z - k_0^2\epsilon_{xz} \\ -k_x k_z + k_0\epsilon_{xz} & -k_x^2 + k_0^2\epsilon_{zz} \end{bmatrix} \begin{bmatrix} E_x \\ E_z \end{bmatrix} = 0. \tag{5}$$

The eigenvalues (5) are the propagation constants of plane waves, propagating in the z-direction under fixed k_x. They are evaluated as:

$$k_z^{(1,2)} = \frac{k_x\epsilon_{xz} \pm \sqrt{(\epsilon_{xz}^2 - \epsilon_{xx}\epsilon_{zz})(k_x^2 - k_0^2\epsilon_{zz})}}{\epsilon_{zz}}. \tag{6}$$

Thus, normal wave vector components of waves, propagating upward and downward with respect to interfaces, are different that is caused by the tilted optical axis and nonzero incidence angle. There is a strong difference compared to isotropic media and uniaxial media with anisotropic axes parallel or orthogonal to interfaces, where $k_z^{(1)} = -k_z^{(2)}$.

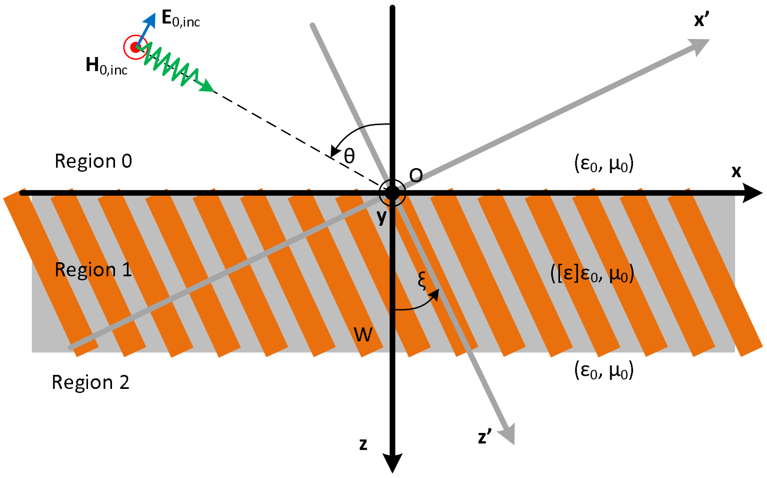

Figure 1: Schematic view of an indefinite medium slab with a tilted optical axis. The z' axis is parallel to the anisotropy axis of MM, the z axis in orthogonal to slab interfaces.

Special case $\xi = -45°,\ \theta = 45°$

Let us consider a special case when $\xi = -45°,\ \theta = 45°$, $\epsilon_\perp = 1$, $\epsilon_\| = -1+\delta$, where $\delta = \alpha+i\beta$, $|\delta| \ll 1$. In this case $\epsilon_{xx} = \epsilon_{zz} = \delta/2$, $\epsilon_{xz} = \epsilon_{zx} = -1+\delta/2$, and $k_x = k_0/\sqrt{2}$. Then we obtain

$$\begin{aligned} k_z^{(1)} &= -k_0/\sqrt{2} \\ k_z^{(2)} &= \frac{3k_0}{\sqrt{2}} - \alpha\frac{2k_0\sqrt{2}}{\alpha^2+\beta^2} + i\beta\frac{2k_0\sqrt{2}}{\alpha^2+\beta^2}. \end{aligned} \quad (7)$$

One can see, that $k_z^{(1)}$ is the same as the z-component of the incident wave in free space. With regard to the wave reflection problem (see Fig. 1), it corresponds to the wave, reflected from the bottom slab interface and its electric field vector is orthogonal to the anisotropy axis. Since $\epsilon_t = 1$, the HM does not affect the propagation of this wave. Contrary, $k_z^{(2)}$ strongly differs from $k_z^{(1)}$ and $|k_z^{(2)}| \to \infty$ if $|\delta| \to 0$. It means that even small material losses ($\beta \ll 1$) cause strong

wave attenuation. For the second wave $E_x/E_z = -k_x/k_z$ if $\epsilon_\perp = 1$, $\epsilon_\| = -1$, so the electric field vector is almost normal to the interface inside the HM. Similar asymmetry appears in any usual anisotropic media with tilted interfaces, but it causes extreme properties for specially designed HM.

1.1 Wave impedance

The transverse wave impedance $Z_{1,2}$ which reads as:

$$Z_{1,2} = \pm\frac{E_x}{H_y} = \frac{\eta}{k_0}\frac{\sqrt{k_x^2 - k_0^2\epsilon_{zz}}}{\sqrt{\epsilon_{xz}^2 - \epsilon_{xx}\epsilon_{zz}}} \tag{8}$$

where $\eta = 120\pi$ Ohm. It is remarkable, that $Z_1 \equiv Z_2$. One can show that if $\xi = -45^\circ$ and $\theta = 45^\circ$ then

$$\begin{array}{c} \epsilon_{xz} = \epsilon_{zx} = \frac{1}{2}(\epsilon_\| - \epsilon_t) \\ \epsilon_{xx} = \epsilon_{zz} = \frac{1}{2}(\epsilon_\| + \epsilon_t). \end{array} \tag{9}$$

So, if $\epsilon_t = 1$ we come to condition of the perfect matching of transverse impedances of the plane wave, incident from free space

$$Z_0 = \eta\frac{\sqrt{k_0^2 - k_x^2}}{k_0} = \eta\frac{1}{\sqrt{2}} \tag{10}$$

and both waves propagating in the HM. This condition does not depend on the frequency while $\epsilon_t = 1$. We will show below that dependence of ϵ_t on the wavelength is a weak for silicon nanowire composites in the interested range. Then, taking into account that $|k_z^{(2)}| \to \infty$, we come to condition of the perfect absorption if the HM possesses low losses. In this case the wave, propagating in the slab with a very short wavelength attenuates at an ultra-short distance.

2 Effective medium model for a silicon nanowire composite

As the model of heavily doped silicon we will use the following Drude formula [1]:

$$\epsilon_i(\omega) = \epsilon_\infty - \frac{\omega_p^2}{\omega(\omega + i\gamma)} \tag{11}$$

where $\epsilon_\infty \approx 11.6$ is the high-frequency limit value of the permittivity [2], and γ is the scattering rate. The plasma frequency and scattering rate are expressed as $\omega_p = \sqrt{Ne^2/(m^*\epsilon_0)}$ and $\gamma = e/(m^*\mu)$, respectively, where e is the electron charge, N is the carrier concentration, m^* is the carrier effective mass, and μ is the mobility. For n-type heavily doped Si the mobility expression is given as [1]

$$\mu = \mu_1 + \frac{\mu_{\max} - \mu_1}{1 + (N/C_r)^\alpha} - \frac{\mu_2}{1 + (C_s/N)^\beta}. \tag{12}$$

Here $m^* = 0.27m_0$, where m_0 is the electron mass, $\mu_1 = 68.5$ cm^2/V s, $\mu_{\max} = 1414$ cm^2/V s, $\mu_2 = 56.1$ cm^2/V s, $C_r = 9.2 \times 10^{17}$ cm^{-3}, $C_s = 3.42 \times 10^{20}$ cm^{-3}, $\alpha = 0.711$ and $\beta = 1.98$. For these parameters $\omega_p = 1.084 \times 10^{15}$ rad/s, $\gamma = 8.586 \times 10^{13}$ rad/s. This formula is valid up to concentration $N = 5 \times 10^{21}$ cm^{-3}. However, we will use it for higher concentration because here our purpose is the illustration of idea only.

Let us consider a composite of silicon nanowires with the concentration p. Homogenization gives the following components of the permittivity tensor:

$$\epsilon_\perp = \epsilon_h \frac{1 + \frac{p(\epsilon_i - \epsilon_h)}{\epsilon_i + \epsilon_h}}{1 - \frac{p(\epsilon_i - \epsilon_h)}{\epsilon_i + \epsilon_h}} \tag{13}$$

and

$$\epsilon_\| = p\epsilon_i + (1 - p)\epsilon_h \tag{14}$$

where ϵ_i is the the complex permittivity of bulk silicon (11) and $\epsilon_h = 1$ is the permittivity of host medium. A special of interest for us is the case when permittivity tensor components satisfy the indefinite medium conditions [3] (Re($\epsilon_\perp$)$\simeq 1$, Re($\epsilon_\|$)$\simeq -1$). Fig. 2 illustrates a possibility to make a Si NW composite, whose permittivity tensor components are close to required ones in the visible range.

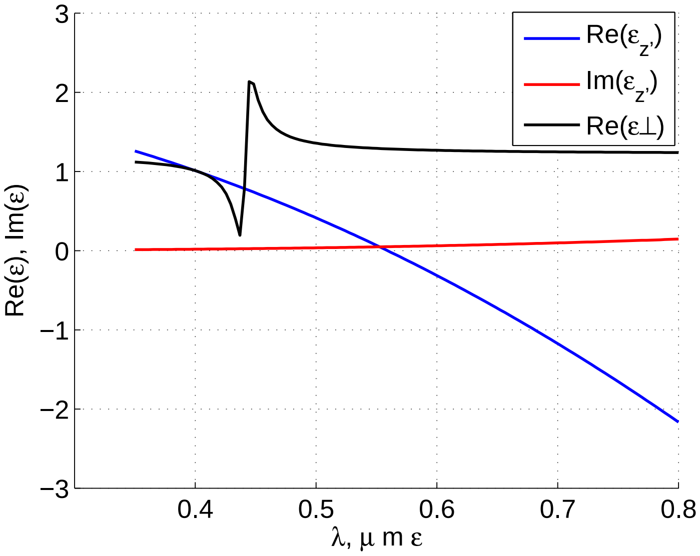

Figure 2: Imaginary part of $\epsilon_{\|}$ (red), real parts of $\epsilon_{\|}$ (blue) and $\epsilon_{\perp}$ (black), respectively. Concentration of Si nanowires in the composite $p = 0.1$, concentration of carriers in doped Si $N = 2 \times 10^{22}\,\mathrm{cm}^{-3}$.

3 Effective medium model for a metal-dielectric multilayer

Let us consider a layered structure consisting of metal (silver) and dielectric alternating layers. Following the effective medium model [4] such materials can be described as uniaxial anisotropic media. The permittivity tensor component $\epsilon_{\|}$, corresponding to polarization parallel to the axis (orthogonal to the layers) is a positive, and the component $\epsilon_{\perp}$ corresponding to polarization orthogonal to the axis (parallel to the layers) is a negative. Formulas for effective parameters look as [4]

$$\epsilon_{\perp} = \frac{h_d \epsilon_d + h_m \epsilon_m}{h_d + h_m} \qquad \frac{1}{\epsilon_{\|}} = \frac{1}{h_d + h_m}\left(\frac{h_d}{\epsilon_d} + \frac{h_m}{\epsilon_m}\right) \tag{15}$$

where h_d and ϵ_d are the thickness and the permittivity of dielectric layers, respectively; h_m and ϵ_m are the thickness and the permittivity of metal layers, respectively. Fig. 3 illustrates a possibility to get needed parameters ($\epsilon_{\perp} < 0$, $\epsilon_{\|} \simeq -\epsilon_{\perp}$) for the metal-dielectric multilayer. Permittivity of silver was taken from [6].

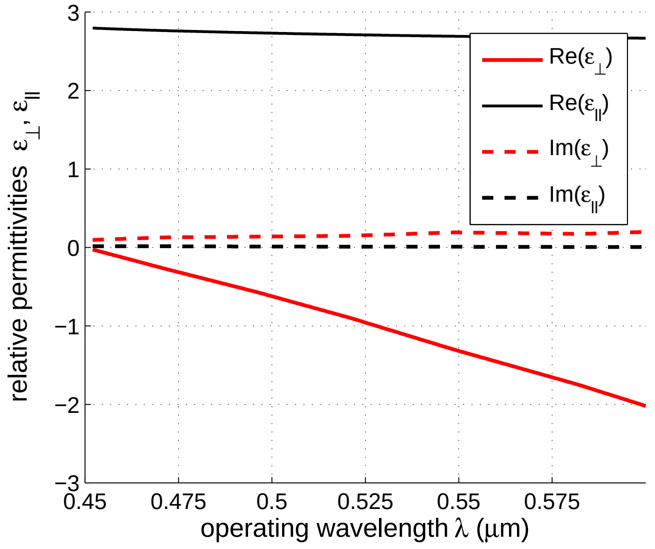

Figure 3: Relative permittivities $\epsilon_\perp$, $\epsilon_{||}$ versus operating wavelength. Parameters of the structure: $h_m = 2\,\mathrm{nm}$, $h_d = 75\,\mathrm{nm}$, $\epsilon_d = 2$.

4 Plane wave transmission through a slab of AHM

For solution of the wave reflection problem we will use the 2×2 transfer matrix method, modified for the general case $|k_z^1| \neq |k_z^2|$. Propagation constants and transverse wave impedances of the waves within the slab can be expressed via elements of the transfer matrix $[M]$ as [5]:

$$\begin{array}{l} e^{ik_z^{(1,2)}W} = \frac{M_{11}+M_{22}\pm\sqrt{(M_{11}+M_{22})^2-4|M|}}{2} \\ Z_{1,2} = \frac{M_{12}}{M_{11}-e^{ik_z^{(1,2)}W}} \end{array} \tag{16}$$

where $|M|$ is the determinant of the transfer matrix and W the thickness of the slab. Solving the system of equations 16 with respect to M_{ij} one can obtain expression for the transfer matrix via the wave impedances and wavenumbers as:

$$\overline{\overline{M}} = \begin{bmatrix} M_{11} & M_{12} \\ M_{21} & M_{22} \end{bmatrix} = \begin{bmatrix} \frac{Z_1 e^{ik_z^{(1)}W} - Z_2 e^{ik_z^{(2)}W}}{Z_1 - Z_2} & -Z_1 Z_2 \frac{e^{ik_z^{(1)}W} - e^{ik_z^{(2)}W}}{Z_1 - Z_2} \\ \frac{e^{ik_z^{(1)}W} - e^{ik_z^{(2)}W}}{Z_1 - Z_2} & \frac{Z_2 e^{ik_z^{(1)}W} - Z_1 e^{ik_z^{(2)}W}}{Z_2 - Z_1} \end{bmatrix}. \tag{17}$$

The transmission coefficient T can be calculated using formula

$$T = \frac{2}{M_{11} + M_{22} + M_{12}/Z_0 + M_{21} Z_0} \tag{18}$$

where Z_0 is defined by formula (10). The reflection coefficient reads as

$$R = \frac{M_{11} + M_{12}/Z_0 - M_{21}Z_0 - M_{22}}{M_{11} + M_{22} + M_{12}/Z_0 + M_{21}Z_0} \tag{19}$$

Absorption A is defined as

$$A = 1 - |R|^2 - |T|^2. \tag{20}$$

References and Notes

[1] Basu, S., Lee, B. J. & Zhang, Z. M. Infrared radiative properties of heavily doped silicon at room temperature. *J. of Heat Transfer* **132**, 023301 (2010).

[2] Markuier, F., Joulain, K., Carminati, J.-P. & Greffet, J.-J. Engineering infrared emission properties of silicon in the near field and the far field. *Opt. Commun.* **237**, 379-388 (2004).

[3] Smith, D. R. & Schurig, D. Electromagnetic wave propagation in media with indefinite permittivity and permeability tensors. *Phys. Rev. Lett.* **90**, 077405 (2003).

[4] Wangberg, R., Elser, J., Narimanov, E. E. & Podolskiy, V. A. Nonmagnetic nanocomposites for optical and infrared negative-refractive-index media. *JOSA B* **23**, 498505 (2006).

[5] Pozar, D. M. *Microwave Engineering*, 3Rd Ed: Wiley India Pvt. Ltd., 2009.

[6] Johnson, P. B. & Christy, R. W. Optical Constants of the Noble Metals. *Phys. Rev. B* **6**, 43704379 (1972).
